# Psychiatric disorders in childhood cancer survivors: A retrospective matched cohort study of inpatient hospitalisations and community-based mental health services utilisation in Western Australia

**DOI:** 10.1177/00048674241233871

**Published:** 2024-02-25

**Authors:** Tasnim Abdalla, David B Preen, Jason D Pole, Thomas Walwyn, Max Bulsara, Angela Ives, Catherine S Choong, Jeneva L Ohan

**Affiliations:** 1 Medical School, Faculty of Health and Medical Sciences, The University of Western Australia, Perth, WA, Australia; 2 School of Population and Global Health, Faculty of Health and Medical Sciences, The University of Western Australia, Perth, WA, Australia; 3 Centre for Health Services Research, The University of Queensland, Herston, QLD, Australia; 4 Department of Paediatric and Adolescent Oncology and Haematology, Perth Children’s Hospital, Nedlands, WA, Australia; 5 Institute for Health Research, The University of Notre Dame Australia, Fremantle, WA, Australia; 6 Department of Endocrinology, Perth Children’s Hospital, Nedlands, WA, Australia; 7 School of Psychological Science, The University of Western Australia, Perth, WA, Australia

**Keywords:** Cancer survivors, paediatric cancer, adolescence cancer, mental disorders, health service utilisation

## Abstract

**Objective:**

We examined the impact of long-term mental health outcomes on healthcare services utilisation among childhood cancer survivors in Western Australia using linked hospitalisations and community-based mental healthcare records from 1987 to 2019.

**Method:**

The study cohort included 2977 childhood cancer survivors diagnosed with cancer at age < 18 years in Western Australia from 1982 to 2014 and a matched non-cancer control group of 24,994 individuals. Adjusted hazard ratios of recurrent events were estimated using the Andersen–Gill model. The cumulative burden of events over time was assessed using the method of mean cumulative count. The annual percentage change in events was estimated using the negative binomial regression model.

**Results:**

The results showed higher community-based service contacts (rate/100 person-years: 30.2, 95% confidence interval = [29.7–30.7] vs 22.8, 95% confidence interval = [22.6–22.9]) and hospitalisations (rate/1000 person-years: 14.8, 95% confidence interval = [13.6–16.0] vs 12.7, 95% confidence interval = [12.3–13.1]) in childhood cancer survivors compared to the control group. Childhood cancer survivors had a significantly higher risk of any event (adjusted hazard ratio = 1.5, 95% confidence interval = [1.1–2.0]). The cumulative burden of events increased with time since diagnosis and across age groups. The annual percentage change for hospitalisations and service contacts significantly increased over time (*p* < 0.05). Substance abuse was the leading cause of hospitalisations, while mood/affective and anxiety disorders were common causes of service contacts. Risk factors associated with increased service events included cancer diagnosis at age < 5 years, leukaemia diagnosis, high socioeconomic deprivation, and an attained age of < 18 years.

**Conclusions:**

The elevated utilisation of healthcare services observed among childhood cancer survivors emphasises the need for periodic assessment of psychiatric disorders, particularly in high-risk survivors, to facilitate early management and optimise healthcare resources.

## Introduction

A cancer diagnosis during childhood or adolescence can trigger a stress response and anxiety symptoms (Mavrides and Pao, 2014), with psychological and behavioural consequences varying depending on the cancer type, age at cancer diagnosis, treatment exposures and access to distress management and social support (Kurtz and Abrams, 2010). Although many young cancer survivors appear to adjust well following treatment cessation (Tonorezos et al., 2022), the intensity and types of cancer treatment can disrupt the child’s cognitive and behavioural developmental course (Mavrides and Pao, 2014), resulting in adverse psychological outcomes for some survivors (Friend et al., 2018). Unfortunately, evidence indicates that mental disorders diagnosed early in life can be persistent and recurrent (Otto et al., 2020), resulting in prolonged morbidity that extends into adulthood (Lash et al., 2021). Thus, understanding the impact of a cancer diagnosis in early life on survivors’ long-term mental well-being is critical for effective personalised care and service planning (Pui et al., 2011). This is particularly important given the growing population of survivors that require support and care.

Current follow-up guidelines recommend periodic assessment of psychosocial issues in all survivors of childhood and adolescent cancers (Children’s Oncology Group (COG), 2018). Despite this, many survivors fail to seek or receive target care that meets the follow-up recommendations (Rehman et al., 2022; Signorelli et al., 2017), which can result in a delay in seeking mental healthcare and potentially lead to more severe and/or complex, adverse psychological outcomes. The ascertainment of psychological outcomes in previous studies often relied on self-reported information collection methods (e.g. questionnaires or structured interviews; Abadie et al., 2020; Bagur et al., 2015; Friend et al., 2018; Gianinazzi et al., 2013), which compromised the representativeness and completeness of the reported outcomes (Lash et al., 2021).

Conducting longitudinal cohort studies using linked population data has helped improve the validity of psychological outcome ascertainment by enabling the monitoring of symptoms and disorders over time (Lash et al., 2021) and the identification of factors associated with a heightened risk of psychopathology (Hirota et al., 2022). The predominant feature of existing longitudinal designs is the examination of hospitalisation registrations, which comprise cases of severe mental disorders (Friend et al., 2018). Five studies from Canada, Denmark, Finland and Sweden have focused on examining the burden of specific mental disorders in childhood and adolescent cancer survivors (CCS) using state- or national-based hospitalisation registers (Ahomaki et al., 2015; Frederiksen et al., 2021; Lund et al., 2013; Nathan et al., 2018; Ross et al., 2003). Four of the studies reported an elevated risk of 1.3–1.5-fold in hospitalisation for mental disorders in CCS compared to siblings or matched controls (Ahomaki et al., 2015; Frederiksen et al., 2021; Lund et al., 2013; Nathan et al., 2018). While the fifth study reported a higher but non-significant risk in the survivors compared to the matched controls (Ross et al., 2003). The estimates reported in existing studies were influenced by factors including the examination of the first event within the same diagnostic category (Ahomaki et al., 2015; Lund et al., 2013; Ross et al., 2003), the exclusion of cases diagnosed with a mental disorder 5 years before cancer diagnosis (Frederiksen et al., 2021), the definition of survival threshold (e.g. 1 versus 5 years post-diagnosis) and the unavailability of hospitalisation records for those younger than 16 years (Nathan et al., 2018). Moreover, longitudinal evidence on the patterns of contact with community-based mental health services among CCS is rare (De et al., 2021; Nathan et al., 2018). Two studies have reported a higher number of outpatient contacts with general practitioners and psychiatrists in survivors of childhood (aged < 18 years; Nathan et al., 2018) and adolescent (aged 15–21 years; De et al., 2021) cancers. Minimal data on utilisation patterns among adolescent survivors (De et al., 2021; Nathan et al., 2018) were reported in these two studies.

In Australia, evidence of the burden of mental health morbidity on CCS and healthcare services are not available. This study aimed to improve our understanding of the burden of mental disorders in CCS in Western Australia (WA) compared to non-cancer controls, with a particular focus on the longitudinal patterns of repeated inpatient hospitalisations and community mental health service contacts, and the contributing risk factors.

## Methods

### Study design

A retrospective examination of whole-population inpatient separations (hereafter referred to as hospitalisations) and community-based mental health service records (hereafter referred to as service contacts) was conducted. Hospitalisation and service contact records were linked through the WA Data Linkage System using a multifaceted process that incorporates probabilistic matching (Supplemental page 4) and a clerical review, to minimise the likelihood of linkage errors (Eitelhuber et al., 2018).

### Study participants

The cancer cases were identified using the WA Cancer Registry (WACR) and the Perth Children’s Hospital (PCH) Oncology Dataset. The WACR is a statutory repository that collects diagnostic information of histologically confirmed, notifiable cancer cases in WA (Government of Western Australia, 2011). The PCH Oncology Dataset is administered by the Department of Paediatric and Adolescent Oncology and Haematology. PCH is the tertiary referral centre for all paediatric and adolescent cancers diagnosed in WA. The diagnostic details (including diagnosis date and age, tumour site and morphology) and demographic characteristics of all cases (aged < 18) diagnosed between 1982 and 2014 were retrieved from the WACR. The tumour site and morphology details were used to assign survivors into major diagnostic groups, according to the International Classification of Childhood Cancer, third edition (Steliarova-Foucher et al., 2005). The PCH Oncology Dataset was used to extract records of non-notifiable cases of Langerhans Cell Histiocytosis (LCH). The identified cancer cases were matched to controls from the WA Birth Registrations with no history of childhood cancer and who were alive at the corresponding CCS’s primary diagnosis date by age (birth month and year) and sex using a 1:10 matching ratio. The Death Registrations records were accessed to determine the survival status at the beginning of the follow-up (i.e. in 1987, which marks the 5-year survival threshold for the earliest diagnosed case) and the cessation of follow-up due to confirmed death. The data flow chart further describes the participant’s selection process, including the exclusion criteria (see Supplemental Figure S1).

### Outcome measures and data sources

The primary outcomes were hospitalisations or service contacts for a clinically confirmed mental disorder or a psychological symptom. The primary outcomes were defined using the Hospital Morbidity Data Collection (HMDC) and Mental Health Information System (MHIS).

The HMDC was used to obtain hospitalisation records from birth to June 2019. The HMDC collects records of all admitted activity in public and private hospitals in WA under the Health Services Act 2016 (Health Department of Western Australia, 2016). Administrative and clinical details (including admission and discharge dates, principal and 21 additional diagnoses fields and funding source) were retrieved. The MHIS was used to obtain details of contact with public-specialised mental health services from birth to December 2019. The MHIS collects records of contact with public ambulatory/non-admitted community-based and specialised inpatient-based services for mental health under the Mental Health Services Act 2014 (Mental Health Commission, 2014). Service contact details related to direct care (i.e. a client or associate of the client is present) or indirect care deemed clinically significant by the mental health specialist are documented (Western Australian Department of Health, 2022). The MHIS also captures the client’s movement across the triage/preassessment and active treatment stages. Thus, the dataset may contain records of clients with clinically confirmed disorders and those with no mention of a symptom or disorder. The principal diagnosis (chiefly responsible for occasioning the episode of care) in the HMDC, and the admission (i.e. service event’s activation) and discharge (service event’s deactivation) diagnosis fields in the MHIS were used to identify records with a mention of a clinically confirmed mental disorder. Mental disorders were coded using the International Classification of Diseases and Related Health Problems, Ninth/Tenth Revision, Clinical/Australian Modification (Independent Health and Aged Care Pricing Authority (IHACPA), 2023; National Center for Health Statistics (NCHS) and Centers for Disease Control and Prevention (CDC), 2021), and classified into seven diagnostic categories defined as psychotic, mood/affective, anxiety, stress, substance abuse, personality and other disorders (Supplemental Table S1).

Hospitalisation records with an interhospital transfer indicator were combined to avoid double-counting diagnoses within the same episode of care. Records of service contacts with the same date were combined to avoid double-counting events and diagnoses. In addition, inpatient records within MHIS were not considered community service contacts.

### Follow-up

The ascertainment of primary outcomes commenced 5 years after the primary cancer diagnosis date (i.e. index date). In the case of survivors with multiple childhood cancers diagnosed within 5 years, the latest cancer diagnosis date was defined as the index date to ensure the acute effects of new treatments are not defined as post-survival effects. The latest diagnosed cancer was used in diagnosis-specific analyses. The corresponding CCS diagnosis date was used as the matched controls’ index date for the start of follow-up to ensure comparable follow-up times. The matched controls were not censored at the time of their corresponding survivor censoring to avoid bias due to loss of follow-up (Lash et al., 2021). It was assumed that all participants remained in the state for the duration of follow-up, based on historical and recent migration trends indicating low out-of-state migration (Australian Bureau of Statistics (ABS), 2020; Clark et al., 2010). The follow-up period extended until 30 June 2019 (for hospitalisations) or 1 December 2019 (for service contacts), or until the date of death, whichever occurred first.

### Covariates

The effect of treatment intensity was accounted for using the cancer diagnosis decade (the 1980s, 1990s, 2000s and 2010s) as a proxy for changes in treatment protocols. The long-term influence of mental disorders (Mulraney et al., 2021) was accounted for using a yes/no indicator for the existence of a mental illness diagnosis/symptom before the primary cancer diagnosis. The potential association between physical comorbidity and hospitalisation for mental disorders (Gentil et al., 2021; Šprah et al., 2017) was accounted for by adjusting for the Charlson comorbidity index (CCI) score (Hude et al., 2011). The CCI score at the time of hospitalisation was calculated using the codes recorded in 21 additional diagnosis fields, which capture current co-existing conditions that influence the required treatments and resources during inpatient care (Government of Western Australia, 2019). The effect of socioeconomic disparities was assessed using the Index of Relative Socioeconomic Disadvantage (IRSD), which assigns individuals into five quintiles (ranging from the most [quintile 1] to the least (quintile 5) disadvantaged) based on the economic and social conditions of people and all households within an area (approximately 250 households within a Statistical Local Area 1) (Australian Bureau of Statistics (ABS), 2018b). The effect of proximity to hospital-based services was assessed using the Australian Statistical Geographic Standard Remoteness Area (RA; Australian Bureau of Statistics (ABS), 2018a), which assigns participants into five residential RAs (major city, inner regional, outer regional, remote and very remote). The IRSD and RA scores were assigned at the start of the follow-up and were assumed to remain unchanged throughout the follow-up. The mental health disparity between Indigenous and non-Indigenous Australians (Australian Institute of Health and Welfare (AIHW), 2023) was accounted for by adjusting for the Aboriginal status of participants. The Aboriginal status was defined using the Aboriginal and Torres Strait status flag, which is determined using a validated algorithm that collects ethnicity data from Government data collections (Eitelhuber et al., 2018). The influence of Medicare beneficiaries (i.e. access to free treatment at public hospitals through a universal public health insurance scheme) and private insurance on hospitalisation frequency was assessed by adjusting for the funding source (public, private insurance/self-funding or others). The matching variables month and year of birth and sex (M/F) were also added to account for residual confounding (Pearce, 2016). Age was added linearly in adjusted models, with the squared term added to account for the non-linear effect of age.

### Statistical analysis

Participants characteristics were summarised as counts and percentages for categorical variables and mean (standard deviation, SD) or median (interquartile range, IQR) for continuous variables. The Mann–Whitney test was used to assess the difference in the length of hospital stay. The hospitalisation period was defined as the time difference between the date of admission and separation, with one day assigned for same-day discharge. The hospitalisation and service contact rates were calculated per 1000 and 100 person-years, respectively, across subgroups. The Andersen and Gill model (Amorim and Cai, 2015) for recurrent events (an extension of the Cox regression model) was used to assess the risk of recurrent hospitalisations and service contacts in survivors compared to controls. The model estimated the hazard ratio (HR) with 95% confidence intervals (CIs), adjusted for the covariates of sex, cancer diagnosis decade (or calendar date), age, age-squared terms, prior history of mental health events, IRSD, RA, Indigenous status and comorbidity. The mean cumulative count method was used to examine the cumulative number of mental health-related events per individual (Dong et al., 2015). The cumulative count of events was presented graphically by time since index cancer diagnosis (or equivalent calendar date) and age, in all survivors and within subgroups defined by the cancer diagnosis decade. The negative binomial regression model was used to calculate the annual percentage change (APC) in hospitalisations and service contacts for any mental disorder over the follow-up period. The regression models were adjusted for sex, age, age-squared term, comorbidity (hospitalisations only), IRSD, RA, prior history to a mental disorder, number of childhood cancer diagnoses and cancer diagnosis decade (or equivalent calendar date).

The analyses were performed using SPSS 26 (IBM Corporation, New York), Stata-MP 17 (College Station, Texas) and R 4.1.2 (R Foundation for Statistical Computing, Vienna). Ethical approvals were granted by the Human Research Ethics Committees at the WA Department of Health, Child and Adolescent Health Service and the University of WA (references: RGS0000001488; RA/4/20/5340). The access to de-identified research data was approved under a legal waiver of consent, as permitted by HRECs and the National Health and Medical Research Council guidelines.

## Results

The study cohort comprised 2977 5-year CCS and 24,994 matched control group participants, followed for a total of 39,825 and 337,563 person-years, respectively. The mean age at the start of follow-up was 13.9 (SD, 5.9) among survivors and 13.7 (SD, 5.9) among controls. The mean age at the end of the study was 27.3 (SD, 10.7) among survivors and 27.2 (SD, 10.6) among controls. Prior to cancer diagnosis, 2.3% of survivors and 2.6% of the control group had a mental disorder diagnosis (see Table 1). The median follow-up duration was 12.3 years (IQR = 5.8–19.9) for survivors and 12.4 years (IQR = 6.0–20.0) for controls. At the end of the follow-up, 95.1% of survivors and 99.3% of controls were alive.

**Table 1.** Clinical and sociodemographic characteristics of childhood cancer survivors and the matched controls, 1982–2014.

Characteristics	Cancer survivors	Matched controls
Total	2977	24,994
Age at cohort entry, mean (SD)	13.9 (5.9)	13.7 (5.9)
Follow-up duration, median (IQR)	12.3 (5.8–19.9)	12.4 (6.0–20.0)
Sex, *n* (%)		
Male	1527 (51.3)	12,890 (51.6)
Female	1450 (48.7)	12,104 (48.4)
Socioeconomic quintile^a^, *n* (%)		
0–20% (most disadvantaged)	571 (19.2)	4807 (19.2)
20–40%	546 (18.3)	4746 (19.0)
40–60%	572 (19.2)	4916 (19.7)
60–80%	591 (19.9)	4948 (19.8)
80–100% (least disadvantaged)	585 (19.7)	5097 (20.4)
Missing	112 (3.8)	480 (1.9)
Residential remoteness^b^, *n* (%)		
Major cities	2044 (68.7)	15,360 (61.5)
Inner regional	312 (10.5)	2594 (10.4)
Outer regional	263 (8.8)	2467 (9.9)
Remote	140 (4.7)	1589 (6.4)
Very remote	73 (2.5)	910 (3.6)
Missing	145 (4.9)	2074 (8.3)
Indigenous status		
Non-Indigenous	2838 (95.3)	23,453 (93.8)
Indigenous	139 (4.7)	1541 (6.2)
Diagnosis period, *n* (%)		
1982–1989	491 (16.5)	–
1990–1999	760 (25.5)	–
2000–2009	1135 (38.1)	–
2010–2014	591 (19.9)	–
Cancer diagnosis age, *n* (%)		
< 5 years	960 (32.2)	–
5–9 years	537 (18.0)	–
10–14 years	714 (24.0)	–
15 to < 18 years	766 (25.7)	–
Index cancer diagnosis type^c^, *n* (%)		
Leukaemia	628 (21.1)	–
Lymphoma	315 (10.6)	–
CNS tumours	428 (14.4)	–
Neuroblastoma	123 (4.1)	–
Retinoblastoma	64 (2.1)	–
Renal tumours	126 (4.2)	–
Hepatic tumours	18 (0.6)	–
Malignant bone tumours	127 (4.3)	–
Soft tissue tumours	213 (7.2)	–
Germ cell tumours	147 (4.9)	–
Other epithelial and melanomas	725 (24.4)	–
Other and unspecified tumours	20 (0.7)	–
Langerhans cell histiocytosis	36 (1.2)	–
Unknown	7 (0.2)	–
Relapse/secondary neoplasm, *n* (%)		
No	2948 (99.0)	–
Yes	29 (1.0)	–
Prior history of mental disorder diagnosis^d^		
No	2909 (97.7)	24,349 (97.4)
Yes	68 (2.3)	645 (2.6)
Vital status at the end follow-up, *n* (%)		
Alive	2832 (95.1)	24,817 (99.3)
Deceased	145 (4.9)	177 (0.7)

SD: standard deviation; IQR: interquartile range; CNS: central nervous system.

a Socioeconomic status was classified according to the Index of Relative Socioeconomic Disadvantage.

b Residential remoteness was classified according to the Australian Statistical Geographic Standard Remoteness Area.

c Index cancer diagnosis was coded according to the International Classification of Childhood Cancer (3rd Edition).

d Any diagnosis of a mental disorder illness before the initial primary cancer diagnosis.

### Any mental health-related service event

**Figure 1.**
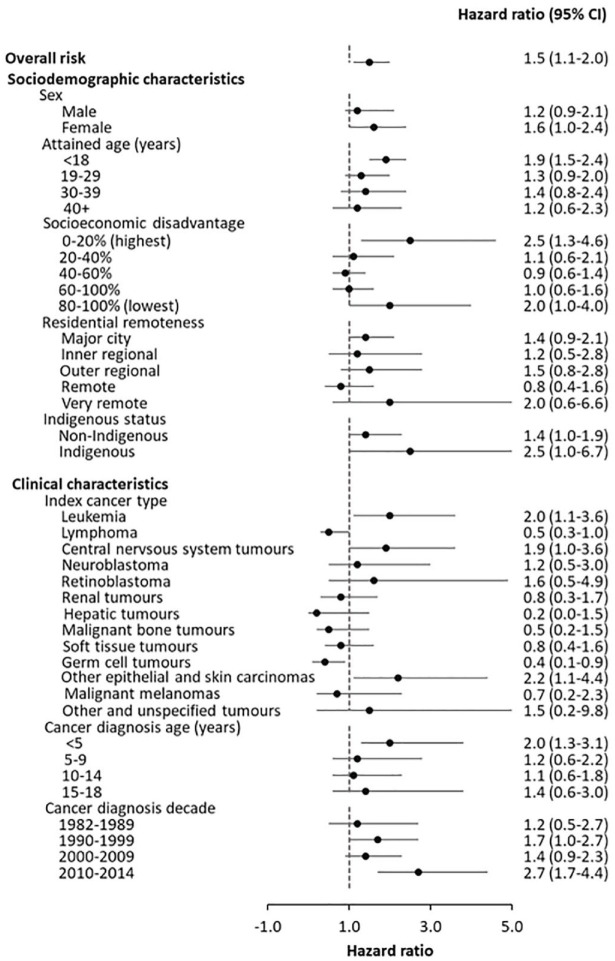
The adjusted hazard ratio of contact with inpatient or community-based mental health services for any mental disorders, in childhood cancer survivors compared to the matched control group, 1987–2019. Adjusted variables included sex, cancer diagnosis decade (or calendar date), age, age-squared terms, a prior history of mental health event, socioeconomic status, residential remoteness, Indigenous status and comorbidity.

Over the follow-up period, 19.8% of survivors (*n* = 589) and 12.6% of controls (*n* = 3141) had at least one hospitalisation or service contact. The average number of mental health-related events per unique individual was 21 among survivors and 26 among controls. The overall rate of any event (per 100 person-years) was 31.7 (95% CI = [31.1–32.2]) in survivors and 24.0 (95% CI = [23.8–24.2]) in controls. The risk of any event was significantly higher in survivors than in controls (HR = 1.5, 95% CI = [1.1–2.0]). Factors associated with a significantly increased risk of a mental health event in survivors included attained age < 18 years (1.9, 95% CI = [1.5–2.4]), high socioeconomic disadvantage (2.5, 95% CI = [1.3–4.6]), leukaemia diagnosis (2.0, 95% CI = [1.1–3.6]), other epithelial and skin carcinomas (2.2, 95% CI = [1.1–4.4]) and cancer diagnosis at age < 5 years (2.0, 95% CI = [1.3–3.0]; see Figure 1). Survivors with an existing mental disorder at cancer diagnosis had a higher rate of any event (rate/100 person-years 178.7, 95% CI = [169.7–188.1]) compared to those without an existing disorder (28.6, 95% CI = [28.1–29.1]) and a significantly higher HR (4.6, 95% CI = [1.2–17.8], *p* < 0.05).

**Figure 2.**
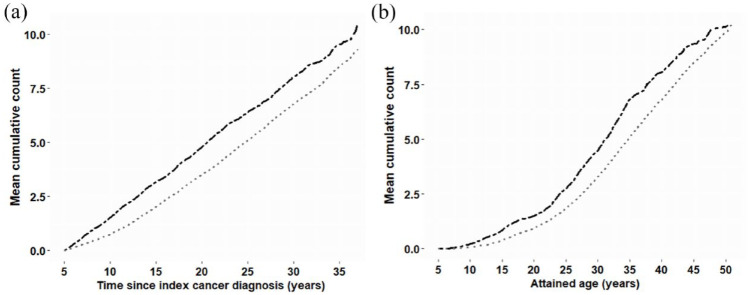
The cumulative burden of contact with inpatient and community-based mental health services for mental disorders, in childhood cancer survivors and matched controls, by (A) time since index cancer diagnosis and (B) attained age.

Survivors experienced a moderately higher cumulative burden of mental health service events per individual with increased time since cancer diagnosis (see Figure 2(A)). Similarly, the overall burden of any mental health event moderately increased with age, particularly after age 20 years. (Figure 2(B)).

### Community-based service contacts

A higher proportion of survivors had at least one service contact than controls (18.4% vs 11.5%, *p* < 0.05). The rate of service contacts (per 100 person-years) was higher in survivors than in controls (30.2, 95% CI = [29.7–30.7] vs 22.8, 95% CI = [22.6–22.9], *p* < 0.05), but the HR for a service contact was not statistically significantly higher in survivors (1.4, 95% CI = [1.0–1.9], *p* > 0.05). The APC in contacts increased by 26.6% (*p* < 0.05) in survivors and by 20.5% (*p* < 0.05) in controls over the follow-up period. The most common causes of service contacts in survivors and their controls were anxiety (10.6%, 95% CI = [8.1–13.4%] vs 13.6%, 95% CI = [12.4–14.9%]), stress (10.2%, 7.8–13.0% vs 9.6%, 95% CI = [8.6–10.8%]) and mood/affective disorders (9.7%, 95% CI = [7.3–12.4%] vs 9.1%, 95% CI = [8.1–10.2%]; see Supplemental Table S2).

In survivors, highest rates of service contacts were observed in females (34.2, 95% CI = [33.4–35.1]), 30–39 years age group (37.9, 95% CI = [36.5–39.3]), high socioeconomic disadvantage (56.9, 95% CI = [55.3–58.6]) and residency in outer regional (33.4, 95% CI = [31.5–35.3]) and metropolitan (32.5, 95% CI = [31.8–33.2]) areas (see Table 2). Clinical factors linked to highest rates of service contacts included a diagnosis with other epithelial and skin carcinomas (49.8, 95% CI = [48.3–51.4]), central nervous system (CNS) tumours (45.0, 95% CI = [43.2–46.7]), leukaemia (34.7, 95% CI = [33.5–36.0]), a cancer diagnosis at age < 5 years (35.3, 95% CI = [34.3–36.4]) or 15–18 years (32.6, 95% CI = [31.5–33.7]). The high rate observed in those diagnosed with primary cancer in 2010–14 (53.3, 95% CI = [49.7–57.2]) is likely to be influenced by the low number of survivors who experienced an event and the short person-time at risk.

**Table 2.** Rate of inpatient hospitalisations and contact with community mental health services in childhood cancer for mental disorders, in childhood cancer survivors and their matched controls, 1987–2019.

Characteristics	Community service contacts				Inpatient hospitalisations			
	Cancer survivors		Matched controls		Cancer survivors		Matched controls	
	No.	Rate/100	No.	Rate/100	No.	Rate/1000	No.	Rate/1000
		(95% CI)		(95% CI)		(95% CI)		(95% CI)
Total	12,025	30.2 (29.7–30.7)	76,862	22.8 (22.6–22.9)	571	14.8 (13.6–16.0)	4153	12.7 (12.3–13.1)
Sex								
Male	5301	26.3 (25.6–27.0)	40,260	23.3 (23.0–23.5)	304	15.5 (13.9–17.4)	2027	12.1 (11.6–12.7)
Female	6724	34.2 (33.4–35.1)	36,602	22.2 (22.0–22.5)	267	14.0 (12.4–15.8)	2126	13.3 (12.8–13.9)
Attained age (years)								
< 18	3573	29.3 (28.4–30.3)	17,373	16.2 (15.9–16.4)	68	5.7 (4.5–7.2)	586	5.6 (5.2–6.1)
19–29	4962	28.1 (27.3–28.9)	32,115	21.7 (21.4–21.9)	290	16.8 (15.0–18.9)	2201	15.3 (14.7–15.9)
30–39	2939	37.9 (36.5–39.3)	21,621	33.6 (33.2–34.1)	157	21.1 (18.1–24.7)	1127	18.4 (17.3–19.5)
40+	551	24.7 (22.7–26.9)	5753	32.6 (31.7–33.4)	56	27.2 (20.9–35.3)	239	14.7 (12.9–16.7)
Socioeconomic quintile^a^								
0–20% (most disadvantaged)	4655	56.9 (55.3–58.6)	14,631	23.1 (22.7–23.5)	180	22.6 (19.6–26.2)	653	9.8 (9.0–10.5)
20–40%	2543	33.6 (32.3–34.9)	20,566	31.8 (31.4–32.3)	141	19.2 (16.2–22.6)	692	10.9 (10.1–11.7)
40–60%	1549	21.4 (20.4–22.5)	16,893	25.2 (24.8–25.6)	68	9.7 (7.7–12.3)	817	12.6 (11.7–13.5)
60–80%	1183	16.3 (15.4–17.3)	10,643	16.2 (15.9–16.5)	66	9.4 (7.4–12.0)	933	15.0 (14.0–15.9)
80–100% (least disadvantaged)	1993	24.5 (23.4–25.5)	8963	13.0 (12.7–13.2)	110	13.9 (11.5–16.7)	850	13.9 (13.0–14.8)
Residential remoteness^b^								
Major city	8931	32.5 (31.8–33.2)	52,317	25.3 (25.0–25.5)	410	15.4 (14.0–16.9)	2732	13.6 (13.1–14.2)
Inner regional	1174	27.5 (26.0–29.1)	8288	23.0 (22.5–23.5)	44	10.5 (9.6–11.6)	508	14.6 (13.4–15.9)
Outer regional	1202	33.4 (31.5–35.3)	7966	22.8 (22.3–23.4)	93	26.6 (21.7–32.6)	426	12.6 (11.5–13.9)
Remote	397	19.8 (18.0–21.9)	5151	24.1 (23.4–24.7)	15	0.8 (0.5–1.3)	330	15.9 (14.3–17.8)
Very remote	315	30.7 (27.5–34.3)	1974	17.5 (16.7–18.3)	9	0.9 (0.5–1.7)	156	14.3 (12.3–16.8)
Indigenous status								
Non-Indigenous	10,263	26.8 (26.2–27.3)	65,816	20.7 (20.6–20.9)	501	13.5 (12.3–14.7)	3540	11.5 (11.1–11.9)
Indigenous	1762	120.1 (114.0–125.8)	–	55.9 (54.9–57.0)	70	49.6 (39.2–62.7)	613	32.1 (29.6–34.7)
Pre-existing mental disorder diagnosis								
No	10,614	27.2 (26.7–27.7)	68,725	20.8 (20.7–21.0)	530	14.0 (12.9–15.2)	3800	11.9 (11.5–12.3)
Yes	1411	173.7 (164.8–183.0)	8137	103.4 (101.2–105.7)	41	52.1 (38.4–70.8)	353	46.5 (41.9–51.6)
Index cancer diagnosis type^c^								
Leukaemia	2940	34.7 (33.5–36.0)	–	–	102	12.4 (10.2–15.1)	–	–
Lymphoma	420	10.6 (9.6–11.6)	–	–	34	8.8 (6.3–12.4)	–	–
CNS tumours	2501	45.0 (43.2–46.7)	–	–	101	18.7 (15.4–22.7)	–	–
Neuroblastoma	380	25.3 (22.9–28.0)	–	–	< 5	NA	–	–
Retinoblastoma	218	23.3 (20.4–26.6)	–	–	< 5	NA	–	–
Renal tumours	263	14.6 (13.0–16.5)	–	–	< 5	NA	–	–
Hepatic tumours	16	7.0 (4.3–11.4)	–	–	< 5	NA	–	–
Malignant bone tumours	143	9.3 (7.9–10.9)	–	–	44	29.5 (22.0–39.7)	–	–
Soft tissue tumours	491	18.7 (17.1–20.4)	–	–	40	15.7 (11.5–21.5)	–	–
Germ cell tumours	223	11.6 (10.1–13.2)	–	–	35	18.7 (13.5–26.1)	–	–
Other epithelial and skin carcinomas	3904	49.8 (48.3–51.4)	–	–	158	20.8 (17.8–24.3)	–	–
Malignant melanomas	452	18.1 (16.5–19.8)	–	–	43	17.6 (13.1–23.7)	–	–
Other and unspecified	28	13.1 (9.0–18.9)	–	–	< 5	NA	–	–
Langerhans Cell Histiocytosis	8	1.3 (0.6–2.5)	–	–	< 5	NA	–	–
Cancer diagnosis age^d^								
< 5 years	4499	35.3 (34.3–36.4)	21,350	18.8 (18.5–19.0)	117	9.5 (7.9–11.4)	901	8.2 (7.7–8.7)
5–9 years	1705	23.3 (22.2–24.4)	13,304	21.2 (20.9–21.6)	61	8.6 (6.7–11.0)	798	13.1 (12.2–14.1)
10–14 years	2512	26.1 (25.1–27.2)	22,211	27.7 (27.3–28.1)	202	21.7 (18.9–24.9)	1332	17.1 (16.2–18.1)
15 to < 18 years	3309	32.6 (31.5–33.7)	19,997	24.7 (24.4–25.1)	191	19.4 (16.8–22.3)	1122	14.3 (13.5–15.2)
Cancer diagnosis decade^e^								
1982–1989	1894	14.7 (14.0–15.3)	17,273	16.6 (16.4–16.9)	171	13.4 (11.6–15.6)	1159	11.3 (10.7–12.0)
1990–1999	5396	38.0 (37.0–39.1)	31,271	25.6 (25.3–25.9)	222	16.0 (14.0–18.2)	1762	14.7 (14.1–15.4)
2000–2009	3954	35.1 (34.0–36.2)	25,417	25.8 (25.5–26.1)	149	13.8 (11.8–16.2)	1151	12.2 (11.5–12.9)
2010–2014	781	53.3 (49.7–57.2)	2901	22.2 (21.4–23.1)	29	23.7 (16.5–34.1)	81	7.4 (6.0–9.2)
Number of childhood cancer diagnoses								
Single diagnosis	12,018	30.4 (29.8–30.9)	–	–	571	14.9 (13.7–16.2)	–	–
Multiple diagnoses	7	2.6 (1.2–5.4)	–	–	–	–	–	–

CI: confidence interval; CNS: central nervous system; NA: not applicable.

a Socioeconomic status classified according to the Index of Relative Socioeconomic Disadvantage.

b Remoteness of residence classified according to the Australian Statistical Geographic Standard Remoteness Area.

c Index cancer diagnosis coded according to the International Classification of Childhood Cancer (3rd Edition).

d Age of matched controls at the time of cancer diagnosis in the corresponding case.

e Calendar period at the time of cancer diagnosis in the corresponding case.

### Inpatient hospitalisations

The overall rate of hospitalisations (per 1000 person-years) for any mental disorder was higher in survivors than in controls (14.8, 95% CI = [13.6–16.0] vs 12.7, 95% CI = [12.3–13.1], *p* < 0.05). A higher proportion of survivors had at least one hospitalisation compared to controls (5.9% vs 4.8%, *p* < 0.05). The APC in hospitalisations increased by 5.5% (*p* < 0.05) in survivors and 6.0% (*p* < 0.05) in controls from 1987 to 2019. The mean length of inpatient days was shorter in survivors (9.3, SD 15.6) than in controls (11.0, SD 26.1); however, the difference was not significant (*p* > 0.05). Of the survivors’ hospitalisations, 82.7% were funded through Medicare, 15.5% through private insurance and self-funds and 0.7% through other funding sources.

In survivors, slightly higher hospitalisation rates were observed in males (15.5, 95% CI = [13.9–17.4]). A higher rate was also observed in those from the most disadvantaged areas (22.6, 95% CI = [19.6–26.2]) and those from outer regional areas (26.6, 95% CI = [21.7–32.6]; see Table 2). Clinical factors associated with higher hospitalisation rates included a cancer diagnosis with a malignant bone tumour (29.5, 95% CI = [22.0–39.7]) and a cancer diagnosis at age 10–14 (21.7, 95% CI = [18.9–24.9]) or 15–18 years (19.4, 95% CI = [16.8–22.3]).

The risk of severe illness necessitating hospitalisation for any mental disorder was 10% higher in survivors than in the control group (HR = 1.1, 95% CI = [0.8–1.4]); however, the risk was not statistically significant. In CCS, the highest hospitalisation rate (per 1000 person-years) was observed for substance abuse (3.6, 95% CI = [3.0–4.2]), followed by mood/affective disorders (2.6, 95% CI = [2.1–3.1]) and psychotic disorders (2.5, 95% CI = [2.1–3.1]; see Table 3). There was no significant difference in the risk between survivors and the control group across all these diagnostic categories. Although the rate of substance abuse disorders (primarily alcohol misuse) was highest in CCS, the proportion of survivors admitted with these disorders was 2.1%.

**Table 3.** Incidence rate and risk of primary hospitalisations for any and specific mental disorders, in childhood cancer survivors and matched controls, 1987–2019.

Diagnostic categories	Cancer survivors		Matched controls		Crude HR (95% CI)	Adjusted HR^a^ (95% CI)
	*N*	Rate^b^ (95% CI)	*N*	Rate^b^ (95% CI)		
All mental disorders	571	14.8 (13.2–16.0)	4153	12.7 (12.3–13.1)	1.2 (0.9–1.6)	1.1 (0.8–1.4)
Psychotic disorders	98	2.5 (2.1–3.1)	617	1.9 (1.7–2.0)	1.3 (0.6–2.8)	1.3 (0.6–2.7)
Mood/affective disorders	100	2.6 (2.1–3.1)	671	2.1 (1.9–2.2)	1.3 (0.6–2.5)	1.1 (0.6–2.2)
Anxiety disorders	65	1.7 (1.3–2.1)	527	1.6 (1.5–1.8)	1.0 (0.7–1.5)	0.9 (0.6–1.3)
Adjustment and stress disorders	61	1.6 (1.2–2.0)	495	1.5 (1.4–1.7)	1.0 (0.7–1.6)	0.9 (0.6–1.4)
Substance abuse disorders	139	3.6 (3.0–4.2)	1154	3.5 (3.3–3.7)	1.0 (0.6–1.6)	1.0 (0.6–1.6)
Other personality disorders	32	0.8 (0.6–1.2)	356	1.1 (1.0–1.2)	0.8 (0.3–1.8)	0.6 (0.3–1.5)
Other mental disorders	76	2.0 (1.6–2.5)	344	1.1 (0.9–1.2)	1.7 (0.9–3.2)	1.6 (0.9–2.8)

HR: hazard ratio; CI: confidence interval.

a HR adjusted for age, an age-squared term, sex, socioeconomic status, residential remoteness, Indigenous status, funding source, cancer diagnosis decade (or equivalent calendar date), a prior history of mental disorder and Charlson comorbidity score.

b Rate/1000 person-years.

## Discussion

This study showed that CCS experienced a higher rate of hospitalisations and community service contacts compared to those with no history of cancer early in life. The overall finding is consistent with the heightened psychiatric morbidity reported in several studies that have investigated the patterns of inpatient services utilisation (Ahomaki et al., 2015; Frederiksen et al., 2021; Lund et al., 2013; Nathan et al., 2018) and outpatient (Nathan et al., 2018) care services for managing psychiatric disorders in CCS.

We performed recurrent event modelling to enable a comprehensive understanding of the demand for inpatient and community-based services. Only two other population-based studies, conducted in Ontario, Canada and Scandinavia (Denmark, Finland and Sweden), have examined recurring mental health-related events in 5-year CCS (Frederiksen et al., 2021; Nathan et al., 2018). Similar to our study, a higher risk of mental health events was observed in the Canadian and Scandinavian CCS cohorts compared to the general population (Frederiksen et al., 2021; Nathan et al., 2018). Our data suggest a 50% statistically significant increase in the overall adjusted risk of seeking inpatient or community-based medical care for psychological issues in survivors compared to general population comparators. The Canadian study reported a 38% statistically significant increase in the overall unadjusted risk of contact with hospital-based (emergency departments and inpatient wards) or community-based (psychiatrists and family physicians) services in CCS than the general population (Nathan et al., 2018). These findings emphasise the need to implement the recommended periodic psychological wellness evaluation (Children’s Oncology Group, 2018) within primary care settings and through other long-term follow-up clinics. Adherence to the recommended annual assessment of psychosocial issues (Children’s Oncology Group, 2018) will enable early detection and referral to community-based services.

We identified a higher mean cumulative number of healthcare events among the individual survivors compared to the controls, which persisted over time and markedly increased beyond the age of 20 years. The observed trend can be partially explained by the association between the accumulation of age-related and treatment-related comorbidities over time and lower psychosocial functioning (Brinkman et al., 2018; Gentil et al., 2021; Šprah et al., 2017). The documented evidence of social, educational and vocational challenges experienced by CCS (Frederiksen et al., 2019), particularly during adolescence (Kosir et al., 2019), and their effect on mental well-being could further explain this finding. The Canadian and Scandinavian studies have reported supporting evidence indicating a higher number of outpatient (Nathan et al., 2018) and inpatient (Frederiksen et al., 2021; Nathan et al., 2018) mental health events in survivors compared to the matched controls across the age spectrum.

We have also identified a significant increase in the annual number of inpatient and community service events among survivors and controls. However, a greater percentage change in community-based service contacts was observed among survivors only, which can be attributed to the potential link between condition severity and observation times (Gasparini et al., 2020). CCS with worse conditions or comorbidities are more likely to interact with other healthcare providers, which might enable various psychological symptoms to be detected and referred for management. The overall trend indicating an annual increase in service provision among survivors and their controls might reflect the impact of the Australian Government’s investment in mental healthcare over the last three decades (Isaacs et al., 2018). Despite implementing the Australian National Mental Health Strategy in 1992, subsequent mental health reform plans targeting early intervention and service access identified unevenly distributed treatment gaps, indicating that many Australians with mental health needs do not seek care (Isaacs et al., 2018). Although national health reform plans have helped improve early intervention and access to services, research shows that many Australians with mental health needs do not seek treatment (Isaacs et al., 2018; Islam et al., 2022; Slade et al., 2009). This suggests that the estimated percentage change in service provision observed among CCS may underestimate this population’s true demand for mental healthcare. Furthermore, increasing service availability does not necessarily ensure effective treatment for individuals who seek care, as evidenced by longitudinal data from Australia, Canada, the United States and England (Jorm et al., 2017). A better understanding of the scale and causes of the treatment gap among survivors is required. In addition, interconnected actions across government sectors are needed to align mental health services with clinical practice guidelines.

We have identified several psychiatric disorders that contributed disproportionally to the need for mental health services, including mood/affective, anxiety, stress, substance abuse and psychotic disorders. The elevated demand for healthcare to manage mood/affective, stress and anxiety disorders can be linked to the neuropsychiatric effects caused by factors including cancer treatments (e.g. adjustment difficulties following exposure to cranial irradiation) and reactions to psychosocial challenges (e.g. dissatisfaction with body image due to treatment-related scarring or disfigurement; Brinkman et al., 2018; Friend et al., 2018; Kosir et al., 2019; Vuotto et al., 2018). While substance abuse (primarily alcohol abuse) and psychotic disorders placed a higher burden on inpatient services in the WA CCS cohort, the adjusted risk of hospitalisations compared to matched individuals did not reach statistical significance. Future research is warranted to explore the prevalence of these disorders using primary care and pharmaceutical dispensing data.

We identified risk factors associated with a significantly higher risk of mental health events among survivors compared to the controls, including a pre-existing mental disorder at cancer diagnosis, leukaemia diagnosis, a cancer diagnosis at < 5 years of age, an attained age of < 18 years and low socioeconomic status. Survivors with a pre-existing mental health illness/symptom at the initial cancer diagnosis were 4.6 times more likely to utilise care services in the post-survival period compared to those with no existing illness/symptom. The cancer diagnosis and its treatments can exacerbate existing psychological issues (Chang and Lai, 2022). Early screening and personalised psychological support during cancer treatment and post-treatment cessation could prevent the progressive deterioration of existing and newly developed disorders.

In survivors of CNS tumours and acute lymphoblastic leukaemia, exposure to therapeutic interventions can increase the risk of developing late neurodevelopmental effects (Macartney et al., 2014), which may manifest as neuropsychological problems (Brinkman et al., 2018). While some studies have reported statistically significant higher psychiatric morbidity in CNS tumour survivors (Ahomaki et al., 2015; Frederiksen et al., 2021; Kosir et al., 2019), we identified an elevated rate but a non-significant adjusted risk between a CNS tumour diagnosis and the rate of mental health service events. Previous studies have also reported evidence of adverse mental health outcomes in survivors diagnosed at age < 5 years (Frederiksen et al., 2021; Lund et al., 2013; Nathan et al., 2018) and in survivors aged < 18 years (Friend et al., 2018; Kosir et al., 2019; Nathan et al., 2018). A finding that is reflective of the heightened vulnerability to distress from cancer diagnosis in young children and the interruption to the cognitive and behavioural development on mental well-being (Mavrides and Pao, 2014). Factors such as parental distress (Macartney et al., 2014) and fear of recurrence can further exacerbate psychological vulnerability in adolescent survivors (Wakefield et al., 2010). The increased risk of psychological problems in CCS from lower socioeconomic status has been linked to cognitive impairments associated with CNS tumour diagnosis and exposure to cranial irradiation and cancer diagnosis at a young age (independent of the primary cancer type), all of which have also been linked to educational and occupational challenges (Frederiksen et al., 2019; Molcho et al., 2019). Evidence also suggests that lower socioeconomic status can contribute to, or be the consequence of, adverse mental health outcomes observed in CCS (Brinkman et al., 2018).

### Strengths and limitations

This analysis is strengthened by using high-quality whole-population cancer and death registrations, which allowed for complete ascertainment of cancer survivors and access to detailed data on all reportable neoplasms. In addition, the completeness of whole-population longitudinal inpatient and community service contacts allowed for assessing the burden of mental disorders in survivors over 32 years of follow-up. The findings reported in this study should be interpreted in the context of several limitations. The lack of detailed diagnostic information on records of community service contacts prevented a more accurate estimation of the cause-specific burden of mental disorders treated in community settings. The lack of detailed treatment and cancer diagnosis stage information on the cancer registry prevented examining the attribution of these factors to the observed mental health outcomes within populations of survivors. Although the findings of the elevated burden of adverse psychological outcomes in CCS align with existing evidence; the estimated effects should be generalised in consideration of differences in the availability of and barriers to mental health services.

In conclusion, our study provides evidence that CCS experience a persistently higher burden of mental healthcare utilisation over time and as they age. It is essential to prioritise early management and treatment of mental disorders across the cancer continuum to prevent expansion in resource utilisation over time. This can be accomplished through ongoing evaluation of survivors’ mental health needs and investment in community-based psychosocial support services, particularly for those with increased vulnerability to psychological challenges.

## Supplemental Material

sj-docx-1-anp-10.1177_00048674241233871 – Supplemental material for Psychiatric disorders in childhood cancer survivors: A retrospective matched cohort study of inpatient hospitalisations and community-based mental health services utilisation in Western Australia Supplemental material, sj-docx-1-anp-10.1177_00048674241233871 for Psychiatric disorders in childhood cancer survivors: A retrospective matched cohort study of inpatient hospitalisations and community-based mental health services utilisation in Western Australia by Tasnim Abdalla, David B Preen, Jason D Pole, Thomas Walwyn, Max Bulsara, Angela Ives, Catherine S Choong and Jeneva L Ohan in Australian & New Zealand Journal of Psychiatry
