# Supplementary material for: Psychiatric disorders in childhood cancer survivors: A retrospective matched cohort study of inpatient hospitalisations and community-based mental health services utilisation in Western Australia
Source: Aust N Z J Psychiatry. 2024 Feb 25;58(6):515–27. doi: 10.1177/00048674241233871 (PMC11128143; doi:10.1177/00048674241233871)
Supplement: sj-docx-1-anp-10.1177_00048674241233871 – Supplemental material for Psychiatric disorders in childhood cancer survivors: A retrospective matched cohort study of inpatient hospitalisations and community-based mental health services utilisation in Western Australia [file sj-docx-1-anp-10.1177_00048674241233871.docx]

**Psychiatric disorders in childhood cancer survivors: a retrospective matched cohort study of inpatient hospitalisations and community-based mental health services utilisation in Western Australia**

Tasnim Abdalla^1^, David B. Preen^2^, Jason D. Pole^3*^, Thomas Walwyn^4,5*^, Max Bulsara^2,6^, Angela Ives^4^, Catherine S. Choong^4,7^, Jeneva L. Ohan^8^

^1^Faculty of Health and Medical Sciences, University of Western Australia
^2^School of Population and Global Health, University of Western Australia
^3^Centre for Health Services Research, The University of Queensland
^4^Medical School, University of Western Australia
^5^Department of Paediatric and Adolescent Oncology and Hematology, Perth Children’s Hospital
^6^Institute for Health Research, The University of Notre Dame Australia
^7^Department of Endocrinology, Perth Children’s Hospital
^8^School of Psychological Science, University of Western Australia

*These authors contributed equally

**Supplementary Materials**

**Supplementary Figure 1**. Data flow chart showing the selection of 5-years childhood cancer survivors and their matched controls

**Supplementary Table 1**. Major diagnostic groups of mental disorders based on the International Classification of Diseases (ICD), 9th (Clinical modification) and 10th (Australian Modification) revisions

**Probabilistic matching method: Western Australia Data Linkage Branch**

**Supplementary Table 2.** The most common causes of contact with community-based mental healthcare services in survivors and their matched controls, Western Australia, 1987-2019


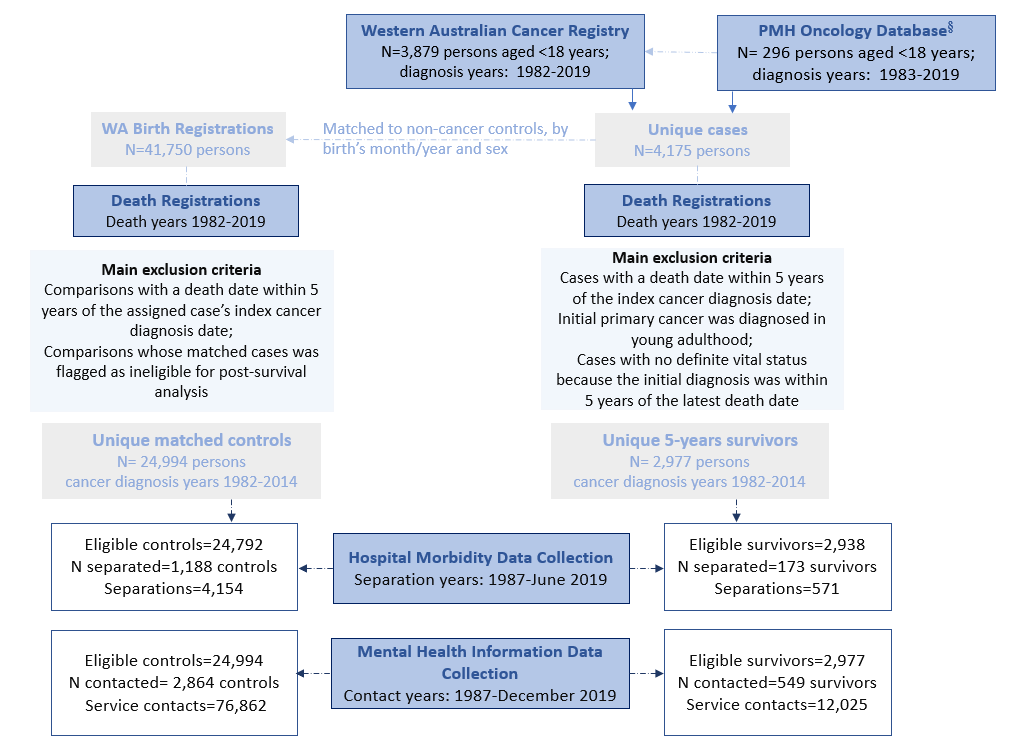


**Supplementary Figure 1.** Data flow chart showing the selection of 5-years childhood cancer survivors and their matched controls. ^§^The dataset used mainly to retrieve cases of Langerhans Cell Histiocytosis.

**Supplementary Table 1.** Major diagnostic groups of mental disorders based on the International Classification of Diseases (ICD), 9th (Clinical Modification) and 10th (Australian Modification) revisions

| **Diagnostic Category** | **ICD-9-CM** | **ICD-10-AM** |
| --- | --- | --- |
| Psychotic disorders (Organic psychosis and schizophrenia) | 290.9, 291 (except 291.2), 292-294 (except 294.1)  295, 297, 298 | F06.0-F06.3  F20 (except F20.4), F21-F25, F28-F29 |
| Mood/affective disorders (includes unipolar & bipolar, with/without psychotic symptoms) | 296 | F30-F34, F38-F39 |
| Anxiety, obsessive compulsive disorder and dissociative disorders.  depression not classified elsewhere. | 300, 311 | F32.0, F32.9, F33.0, F33.3, F34.1, F40-F42, F44, F45.0, F45.1-F45.2, F48 (except F48.0), F63.3, F68.0, F20.4 |
| Stress related disorders (incl. short and long-term disorders). | 308, 309 | F43, F93.0, F94.0 |
| Substance-abuse (psychoactive and non-dependence) disorders | 303-305 | F10-F19, F55 |
| Disorders of childhood and psychological development^§^ | 299, 312-315, | F63 (except 63.3), F80-F84, F88-F94 (except F93.0, F94.0), F98 |
| Personality disorders | 301 | F34.0, F60-F62, F68-F69 |
| Intellectual disorders^§^ | 317-319 | F70-F73, F78-F79 |
| Intentional self-harm | E950-959 | X60-X84, R45.81-Y87.0 |
| Mental health descriptive codes^§^ | 799.2, 368.1 | V11, V15.4, V17.0, V40, V66.3, V67.3, V70.1-V70.3, V71, V79, R45, R45.1, R45.4-R45.8, Z00.4, Z03.2, Z04.6, Z09.3, Z13.3, Z50.2-Z50.4, Z54.3, Z56.6. Z62-Z65, Z70, Z71.4-Z71.6, Z71.9, Z73, Z81.1-Z81.4, Z81.8, Z86.4, Z86.5, Z91.4, F99, T74.3, |
| Other mental health disorders^§^ | 302, 306, 307, 316 | F45, F45.3, F45.4, F45.8, F45.9, F48.0, F50-F54, F59, F64-F66, F95, V400 |

^§^The major diagnostic group ‘*Other mental disorders’* was defined using the codes in these diagnostic categories.

**Probabilistic matching method: Western Australia Data Linkage Branch**

The Western Australia Data Linkage Branch (WA DLB) uses the probabilistic matching method (also known as probabilistic linkage) to identify records that refer to the same individual across different databases because of the lack of a population-wide personal unique identifier that can be used for linkage (Eitelhuber et al., 2018). This method estimates the likelihood that two records match based on the similarity of standard fields or identifiers, such as the given name, surname, date of birth, and address. It uses statistical algorithms to calculate match probabilities, considering the frequency of each identifier and the amount of agreement or disagreement between them (Eitelhuber et al., 2018). The probabilistic matching model used by WA DLB is valid and is subjected to continuous improvement and review of the stability of links over time as new datasets are incorporated (Eitelhuber et al., 2018).

**Reference**

Eitelhuber TW, Thackray J, Hodges S and Alan J (2018) Fit for purpose - developing a software platform to support the modern challenges of data linkage in Western Australia. *International Journal of Population Data Science* 3(3): 435-435.

| **Supplementary Table 2.** The most common causes of contact with community-based mental healthcare services in survivors and their matched controls, Western Australia, 1987-2019 | | | | |
| --- | --- | --- | --- | --- |
| **Diagnostic category^§^** | | **Prevalence (95% CI)** | | |
|  | | Survivors | | Matched controls |
| Anxiety disorders | **10.6% (8.1-13.4)** | | **13.6% (12.4-14.9)** | |
| Stress disorders | **10.2% (7.8-13.0)** | | **9.6% (8.6-10.8)** | |
| Mood/affective disorders | **9.7% (7.3-12.4)** | | **9.1% (8.1-10.2)** | |
| Other mental disorders | 7.3% (5.3-9.8) | | 3.1% (2.5-3.8) | |
| Descriptive mental disorders | 6.0% (4.7-8.3) | | 6.7% (5.8-7.7) | |

^§^The diagnostic categories were identified using the admission and
 discharge diagnosis fields in the Mental Health Information Systems. Refer
 Supplementary Table 1 for the codes used to identify the presence of diagnoses. CI, confidence intervals.
